# Providing food security in Gaza for the “day after”

**DOI:** 10.1186/s13584-025-00700-9

**Published:** 2025-06-10

**Authors:** Elliot M. Berry, Dorit Nitzan, Martin Kussmann

**Affiliations:** 1 Braun School of Public Health, Faculty of Medicine, Hebrew University -Hadassah Medical School, Jerusalem, Israel; 2 School of Public Health, Ben Gurion University of the Negev, Beersheba, Israel; 3 Competence Center for Nutrition, Bavarian Research Organization for Agriculture, Freising, Germany; 4 Kussmann Biotech GmbH, Nordkirchen, Germany

**Keywords:** Food security, Hamas-Israel war, The “day after”, Health for peace, Conflicts

## Abstract

Poverty, conflict and war are the most prominent reasons for food insecurity worldwide including for the population of Gaza since October 7, 2023. It has been shown that at least during the seven-month period between January and July, 2024, an adequate supply of food was delivered to Gaza. However, a distinction must be made between food availability (entering Gaza), and food accessibility (food supply actually reached at the household level). The latter was apparently controlled by Hamas; and there are no reliable data available on the actual distribution of food. A prerequisite for achieving a better “day after” for the population of Gaza depends on achieving a permanent end to the hostilities between Hamas and other Gazan militants with Israel. That must be a top priority for policymakers. Nonetheless, understanding the elements involved in the planning for a successful “day after” can begin now. We know that most of the population needs housing, as well as sufficient, adequate and accessible food, water, energy sources, adequate health services for acute and chronic medical and surgical conditions, mental health, and preventive care. In this article, we focus on planning for food and nutrition security for the “day after,” a process that will require actions along the six dimensions of food security– availability, accessibility, utilization, stability, sustainability, and agency. We outline these dimensions and their necessary components.

## Background

Poverty, conflict and war are the most prominent reasons for food insecurity worldwide [1]. Although vocal concerns about food insecurity and impending famine were raised shortly after Hamas invaded southern Israel on October 7, 2023, taking 251 hostages, followed by Israel’s subsequent retaliatory land and air attacks on Gaza, it should be noted that significant food insecurity in Gaza antedated the current war. A report by Simone Lipkind of the Council on Foreign Relations stated that in 2022, 64.3% of Gaza’s population was already classified as being moderately or severely food insecure, and 77% of residents reported that all of their family members had reduced the number of meals they consume per day [2]. In the 15 ½ months between October 7, 2023, and the beginning of a six-week truce on January 19, 2025, there were major changes in agriculture in Gaza. Before October 7, “agricultural land covered approximately 47% of the Gaza Strip and produced enough food to serve up to a third of local demand…”; but then, “according to the UN, 82% of croplands, 55% of on-farm irrigation systems and 78% of greenhouses have been damaged, leaving once-productive fields barren. Nearly 70% of agricultural wells have been damaged while 96% of cattle and 99% of poultry have died [3].”

A recent paper by Fliss-Isakov, et al., has carefully studied delivery of food to Gaza through Israel between January and July 2024 and demonstrates that the average amount of energy available per person per day was 3,004 kcal, with 98 g of protein (13% of energy), 61 g of fat (18% of energy), and 23 mg of iron [4].” It appears that while food delivery may have varied from day to day, the overall amount of food and its general nature, at least during the seven months studied, was adequate.

There is a distinction between food delivery, availability (entering Gaza), and food accessibility (food supply at the household level). The latter is apparently controlled by powers of which Israel has no control, including Hamas. There are no reliable data available on the actual distribution of food to the household level.

At the time of submission of our article (March 2025), the living conditions for the people in Gaza were most precarious due to the ongoing war and the concomitant damage and destruction of the infrastructure. Since then, as stated in a Policy Paper by Ofer Guterman, published online by the Israel Institute of National Security Studies, Gaza has become largely uninhabitable due to massive or total destruction of the regional infrastructure [5]. This means that secured and civilized life in Gaza has become virtually impossible and even for the most essential means of basic healthcare and sanitation, not to mention education and business activities which can no longer be sustained.

During the six weeks of ceasefire following January 19, 2025, that were supposed to be the first phase of a three-stage process, large amounts of humanitarian aid entered Gaza. Then, Israel felt that negotiations for extending the ceasefire were not fruitful, and in an attempt to get to an appropriate result at the negotiating table, Israel, announced it was halting the entry of aid into Gaza and estimated that the area had a four-to-five-month supply of food for a population of 2.2 million persons. (COGAT Humanitarian Efforts Israel Dashboard. https://gaza-aid-data.gov.il/main/).

Two months later, due to international concern about a threat of famine and international pressure, Israel began to allow food aid to enter Gaza. At the time of this writing, aid has just begun, and the situation going forward is unclear.

The conflict that began on October 7, 2023, has been particularly devastating for Gaza. It compounded pre-existing conditions there and now the population has a large set of very basic needs, especially for ensuring food security [6]. 

Most of the population needs shelter, as well as sufficient food, water, sanitation, hygiene, energy, adequate health care for acute and chronic medical and surgical conditions, including mental health, and preventive care. As the political landscape continues to shift, the necessity for coordinated international humanitarian strategies remains critical to navigate these multisided challenges effectively. At the same time, there are critical questions about how these needs can be addressed to provide a better “day after” the war. In this communication, we focus primarily on establishing food security, and hopefully sustainable food and nutrition security, for the population.

## Food security for the “day after”

There is now an opportunity to propose plans for managing and improving food security for the “day after” and beyond. We believe that the total program of recovery and reconstruction for Gaza should follow the UN Rights Up-front Approach that was instituted in 2013 by then UN Secretary General, Ban Ki-Moon [7]. In this approach, the rights of people are the priority: people have the right to assistance, the right to life with dignity, the right to protection and security, and the right to fully participate in decisions relating to their recovery. All action plans involve consulting with stakeholders to create a Post-Disaster Needs Assessment (PDNA) and prepare a Disaster Recovery Framework (DRF). The European Union, the UN Development Group, and the World Bank have collaborated on the development of guides for conducting Post Disaster Needs Assessments (PDNA) and for preparing Disaster Recovery Frameworks (DRF). “Both guides are based on good practices and experiences from around the world and are intended to coalesce international and local support behind a single, government-led post-disaster recovery process.” The guides can be accessed at: https://www.undp.org/publications/pdna.

The Post-Disaster Needs Assessment (PDNA) and Disaster Recovery Framework (DRF) are internationally recognized methodologies developed by the United Nations, World Bank, and European Union to guide countries through comprehensive post-disaster assessments and structured recovery planning. Recent implementations in El Salvador and Haiti highlight their effectiveness in facilitating coordinated and resilient recovery efforts. Details may be accessed at: [8, 9]

In El Salvador in 2020, the PDNA estimated damages and losses at about USD2.9 billion. The approach addressed immediate recovery needs and laid the groundwork for improved emergency preparedness and risk reduction strategies.

In Haiti, following the 2021 earthquake a DRF provided a roadmap for recovery, focusing on priority sectors and aligning efforts with national development goals. This structured approach enabled Haiti to prioritize interventions, attract international support, and strengthen its institutional frameworks for future emergency management.

Furthermore, to provide food security to the population of Gaza, the PDNA related to nutrition should follow the Integrated Food Security (IPC) Phase Classification. IPC provides strategically relevant information to decision makers that focuses on short-term objectives to prevent, mitigate or decrease severe food insecurity that threatens lives or livelihoods. An introduction to the classification and other IPC materials can be found at: https://www.ipcinfo.org/ipcinfo-website/ipc-overview-and-classification-system/ipc-acute-food-insecurity-classification/en/. From there, one can access the Technical Manual for the IPC [10].

To ensure food security to a population, six aspects or dimensions must be considered. These dimensions are availability, accessibility, utilization, stability, sustainability and agency [11, 12]. These are defined as follows and itemized below:

1. “Availability” refers to the total food supply available in the country, from local production and imports.
2. “Accessibility” refers to the food obtained at the household level.
3. “Utilization” is the food consumed by an individual.
4. “Stability” is the short-term time dimension dealing with the ability to withstand financial, man-made or natural disasters.
5. “Sustainability” is the long-term time dimension referring to the food supply for future generations.
6. “Agency” is the role of the civil society and NGOs in providing food aid.
7. **Availability** of sufficient food at the “national level” for Gaza is likely to entail:

- Ensuring food delivery by sea (building a new Gaza port), by air; by land– via Israel and Egypt.
- Providing at least 150 trucks per day with ready-to-eat high-energy, protein and micronutrients food items.
- Deciding who to supply - for example, giving priority to the most vulnerable groups such as pregnant and lactating women, infants, and the elderly.
- Promoting breastfeeding.
- Guarding storage and refrigerators to prevent theft and vandalism.
- Reviving crop production and animal husbandry (cattle, sheep, goats, poultry).
- Ensuring a stable supply of eggs, dairy products, legumes, vegetable and fruit.
- Building and repairing bakeries.
- Restoring local production, including home-based and community-based approaches.
- Reviving fishing activities.

2. **Accessibility (physical & economic)** at the household level.

The war in Gaza has led to displacement of the majority of the population. Most of the population was living in temporary shelters during the war, and it is likely that, despite efforts by many to return to their homes, many will continue living in such settings. Getting food regularly to households will require planning and professional implementation, while giving attention to the following:

- Camp management and organization to ensure distribution to all in need, following the standards of the SPHERE organization. See: https://www.spherestandards.org/humanitarian-standards/ These standards are designed to uphold the dignity and rights of crisis-affected people through principled, accountable and quality humanitarian action, and involve four categories of services:
  i. water supply, sanitation, and hygiene (WASH).
  ii. food security and nutrition.
  iii. shelter and settlement; and.
  iv. access to health care.
- Improve the infrastructure for street food.
- Cooking and kitchen facilities requiring generators and fuel.
- Food aid (cash, stamps…).
- Provision of seeds, animal feed, and small domestic animals.
- Reopening schools and providing school meal programs.
- Provide information about location and timing of food supplies (including via social media).

3. **Utilization** (food consumption) at the individual level:

- Ensuring careful nutritional rehabilitation to avoid refeeding syndrome among the severely malnourished [13].
- Ensuring food safety.
- Applying public health preventive measures:
  i. Monitoring and prevention of infectious diseases: polio, hepatitis, salmonella, cholera.
  ii. Providing clinics & field hospitals.
  iii. Continuous monitoring of infant nutritional status, growth and development, especially over the period of 0–56 months.
- Ensuring nutritional prescriptions for patients with special requirements, such as diabetes, celiac disease, and others.

4. **Stability** during short term crises or disasters.
  - Developing and enhancing individual and community coping strategies to deal with the many challenges of daily living [14].
  - Providing necessary financial aid according to defined criteria.
  - Encouraging local food production especially when the national food supply is interrupted.
5. **Sustainability** over the long-term:

Food, nutrition, water, and energy security are all critical for sustainability of food security:

- Carrying out a Post-Disaster Needs Assessment (PDNA) with an eye to the long-term future.
- Preparing a Disaster Recovery Framework that considers both what must be done promptly and what has to be done for the future. See: https://www.fema.gov/emergency-managers/national-preparedness/frameworks/recovery.
- Implementing those plans at the local government level.
- Providing employment for the local population.
- Rebuilding the education system.
- Sustaining local production: family- and community-based agriculture (“seeds for health”).
- Rebuilding local food industries.
- Developing fishing.
- Rebuilding the ecosystem and environment.

6. **Agency**.

- Developing civil society involvement to empower the local population to work together on building resilience to food insecurity to achieve food security [15].
- Collaborating with NGOs to coordinate food aid.

Food and nutrition security will have to be restored according to short- and long-term goals. Until there is adequate housing and infrastructure for water and energy, food and sanitation will continue to be on an emergency basis. Establishing availability at the national level will also require multiple routes for entry of aid into Gaza by air, land, and sea. Long-term plans involve restoring the ability of the population to provide a substantial percentage of its own food by reviving agriculture, animal husbandry, and the fishing industry. In addition, the ability to produce food products such as bread and pastry must be enhanced.

In March 2025, Egypt proposed a plan for “Early Recovery, Reconstruction, and Development of Gaza”, which may be accessed at: https://egyptembassy.net/media/Gaza-Recovery-Reconstruction-and-Development-Plan.pdf?utm_source=chatgpt.com.

This includes some elements addressing food security and safety that are included in our approach. Among them: Restoration of critical services, including water supply, electricity, and healthcare facilities; Establishment of water, sewage, and fire prevention systems, including desalination plants, drinking and fire water reservoirs, irrigation water reservoirs, and wastewater treatment plant; and Establishment of a fishing port, a commercial seaport, and Gaza International Airport to boost trade and economic connectivity. This plan is divided into two stages the first to be implemented in the first two years, followed by the second stage.

## Implications for policymakers

In the Background section, we stated a pre-requisite to establishing a successful “day after” will be a permanent end to the hostilities between Hamas and other Gazan militants with Israel. Thus, this becomes the major priority for policymakers.

Although food security is a key element in a better future for the population of Gaza, it should be considered part of general public health rehabilitation measures. The so-called humanitarian-development-peacebuilding (HDP) nexus should provide an important guide for these actions [16]: In 2016, the United Nations proposed “a way of working with the populations affected by crisis to reduce their humanitarian needs by addressing key root causes and decreasing the risks and vulnerabilities they face.” “The HDP nexus strives to enhance the coordination, collaboration and coherence of humanitarian, development and peace actions, particularly in protracted crises and conflict settings.” In the conflict between Israel and Hamas, there is a sincere humanitarian appeal on both sides, to comfort the mourners, rehabilitate the injured and maimed, and return the hostages and internally displaced persons to their homes. Then, and only then, will it be possible to work towards attaining an eventual constructive peace in the region.

In this article, we have focused on the need to establish food security in a way that is sustainable for the future. In an environment of peacebuilding, achieving sustainable food security for Gaza will require international guarantees, not just Israeli guarantees, that take responsibility for the framework, prioritize its components, develop detailed project plans and logistics, and commit sufficient resources to carry out and implement them. The importance of assuring the long-term safety of citizens of both sides cannot be over-emphasized.

One thing is clear: appropriate representatives of the population of Gaza must be included in the future planning. Only legitimate, credible, empowered and peace-oriented representatives on both the Palestinian and Israeli side can break the spiral of mistrust, hate and violence. For this, *interim* international support is needed to run Gaza. More cannot be stated at present, until it is clarified which agencies / governments will be involved in running Gaza and preventing the return of Hamas about which there is a near unanimous international consensus. Despite the many conflicts and suffering over many years, a better life for the people of Gaza and Israel is possible. It is a moral obligation of the international community to help them achieve it.

## Data Availability

No datasets were generated or analysed during the current study.

## Abbreviations

HDP: Humanitarian development-peacebuilding
IPC: Integrated Food Security Phase Classification
NGO: Non-governmental organization
PDNA: Post-Disaster Needs Assessment
UNDP: United Nations Development Programme
